# Vitamin D in plants: a review of occurrence, analysis, and biosynthesis

**DOI:** 10.3389/fpls.2013.00136

**Published:** 2013-05-13

**Authors:** Rie B. Jäpelt, Jette Jakobsen

**Affiliations:** Division of Food Chemistry, National Food Institute, Technical University of DenmarkSøborg, Denmark

**Keywords:** plants, algae, biosynthesis, vitamin D, 25-hydroxy vitamin D, 1, 25-dihydroxy vitamin D, sterols, detection

## Abstract

The major function of vitamin D in vertebrates is maintenance of calcium homeostasis, but vitamin D insufficiency has also been linked to an increased risk of hypertension, autoimmune diseases, diabetes, and cancer. Therefore, there is a growing awareness about vitamin D as a requirement for optimal health. Vitamin D_3_ is synthesized in the skin by a photochemical conversion of provitamin D_3_, but the necessary rays are only emitted all year round in places that lie below a 35° latitude. Unfortunately, very few food sources naturally contain vitamin D and the general population as a results fail to meet the requirements. Fish have the highest natural content of vitamin D expected to derive from an accumulation in the food chain originating from microalgae. Microalgae contain both vitamin D_3_ and provitamin D_3_, which suggests that vitamin D_3_ exist in the plant kingdom and vitamin D_3_ has also been identified in several plant species as a surprise to many. The term vitamin D also includes vitamin D_2_ that is produced in fungi and yeasts by UVB-exposure of provitamin D_2_. Small amounts can be found in plants contaminated with fungi and traditionally only vitamin D_2_ has been considered present in plants. This review summarizes the current knowledge on sterol biosynthesis leading to provitamin D. It also addresses the occurrence of vitamin D and its hydroxylated metabolites in higher plants and in algae and discusses limitations and advantages of analytical methods used in studies of vitamin D and related compounds including recent advances in analytical technologies. Finally, perspectives for a future production of vitamin D biofortified fruits, vegetables, and fish will be presented.

## Introduction

The main function of vitamin D is in maintenance and regulation of calcium levels in the body and vitamin D is, therefore, critically important for the development of a healthy skeleton. Thus, vitamin D insufficiency increases the risk of osteoporosis, but has also been linked to an increased risk of hypertension, autoimmune diseases, diabetes, and cancer (Hyppönen et al., 2001; Cantorna and Mahon, 2004; Holick, 2004; Lappe et al., 2007; Pittas et al., 2007; Kendrick et al., 2009). As a result, there is a growing awareness about vitamin D as a requirement for optimal health. Vitamin D_3_ is synthesized in the skin by a photochemical conversion of provitamin D_3_, but the necessary UVB rays (290–315 nm) are only emitted all year round in places that lie below a 35° latitude. Thereby, a dietary intake of vitamin D becomes essential, but very few food sources naturally contain vitamin D. The consequence of a low dietary intake and limited vitamin D derived from the sun is that the general populations fail to meet their vitamin D requirements (Brot et al., 2001; Bailey et al., 2010). Fish have the highest natural amount of vitamin D_3_, which is expected to derive from a high content of vitamin D_3_ in planktonic microalgae at the base of the food chain (Takeuchi et al., 1991; Sunita Rao and Raghuramulu, 1996a). The occurrence of vitamin D_3_ in algae suggests that vitamin D_3_ may exist in the plant kingdom and vitamin D_3_ has also been identified in several plant species as a surprise to many (Boland et al., 2003). The term vitamin D also includes vitamin D_2_ that is produced in fungi and yeast by UVB-exposure of provitamin D_2_ and small amounts can be found in plants contaminated with fungi. Traditionally, only vitamin D_2_ has been considered present in plants.

Two reviews exist on vitamin D compounds in plants (Boland, 1986; Boland et al., 2003). Boland (1986) focused on plant species with vitamin D-like activity, possible functions of vitamin D_3_ in these plants and metabolism of 1,25(OH)_2_D_3_ glycosides in animals. Boland et al. (2003) dealt with the detection, presence, and tissue distribution of vitamin D_3_ compounds in flowering plants, the production of vitamin D_3_ and derived metabolites in plant cultures, and biological functions of vitamin D_3_ in flowering plants. However, important questions still remain, especially regarding the biosynthesis of vitamin D in plants and the present review, therefore, summarizes current knowledge on sterol biosynthesis leading to provitamin D. Before discussing this subject, essential information on vitamin D synthesis, metabolism, biological functions, as well as dietary sources and recommended intake of vitamin D are described. This review also considers the occurrence of all vitamin D active compounds existing in plants and algae and discusses the advantages and disadvantages of analytical methods applied for studying vitamin D and related compounds including recent advances in analytical technologies. Finally, perspectives for the future production of vitamin D biofortified fruits, vegetables, and fish will be presented.

## Vitamin D

### Synthesis and activation of vitamin D

**Figure 1:**
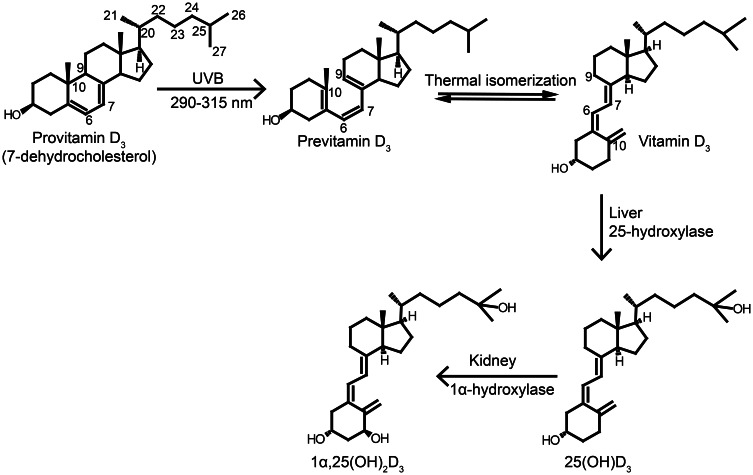
**Synthesis and activation of vitamin D.** Vitamin D_3_ is synthesized in the skin upon UVB exposure. The UVB exposure of provitamin D_3_ (7-dehydrocholesterol) in the skin breaks the B-ring to form previtamin D_3_, which undergoes thermally induced rearrangement to vitamin D_3_. Vitamin D_3_ is transported to the liver where it is hydroxylated at C-25 by the enzyme 25-hydroxylase producing 25OHD_3_, which is the major circulating form in vertebrates. The 25OHD_3_ is hydroxylated a second time at C-1 in the kidneys to the active metabolite 1,25(OH)_2_D_3_. Figure adapted from Jäpelt et al. (2012).

Vitamin D is classified into five different classes numbered 2–6. The two main forms of vitamin D are cholecalciferol (vitamin D_3_) and ergocalciferol (vitamin D_2_), which differ structurally in the side chain, where vitamin D_2_ has a C22–C23 double bound and an additional methyl group at C24 (Figure 1). The vitamins are secosteroids, i.e., steroids with a broken ring. Vitamin D_2_ is produced in fungi and yeast by a UVB-exposure of ergosterol (provitamin D_2_), whereas vitamin D_3_ is produced by UVB-exposure of 7-dehydrocholesterol (provitamin D_3_) in the skin (Figure 1). The conversion to the previtamin D happens by an exposure to sunlight at 290–315 nm (UVB) (Figure 1). Conversion also happens at lower wavelengths, but solar radiation below 290 nm is prevented from reaching the earth's surface by the ozone layer in the stratosphere (MacLaughlin et al., 1982). High-energy photons are absorbed in the conjugated 6,7-diene in the B-ring of ergosterol and 7-dehydrocholesterol, which results in a ring opening at C9 and C10 to form previtamin D (Havinga, 1973). Previtamin D is biologically inactive and thermodynamically unstable and undergoes a transformation to vitamin D in a temperature-dependent manner (Havinga, 1973). Previtamin D_3_ will by prolonged UVB-exposure be converted to the inactive forms lumisterol and tachysterol to protect the organism from vitamin D toxicity (Holick et al., 1981). Synthesis of vitamin D in the skin depends on, e.g., season and latitude. The solar zenith angle increases during the winter months and with latitude. When the solar zenith angle is large, filtration of sunlight through the ozone layer takes place through an increased path length, decreasing the UVB photons that penetrate into the earth's surface. As a result, the rays necessary for the vitamin D production are only emitted all year round in places that lie below 35° latitude (Holick, 2003). In the northern hemisphere this is, e.g., Northern Africa and Los Angeles.

Vitamin D from the skin diffuses into the blood, where it is transported by vitamin D binding protein (DPB) to the liver, whereas vitamin D from the diet is absorbed in the small intestine and transported to the liver via chylomicrons and DBP. Vitamin D is biologically inactive and the activation involves two hydroxylations (Figure 1). First, vitamin D is hydroxylated in the liver at C-25 by a 25-hydroxylase to yield 25-hydroxyvitamin D (25OHD) (Jones et al., 1998; Prosser and Jones, 2004). The activity of 25-hydroxylase is poorly regulated and dependent primarily on the concentration of vitamin D (Bhattacharyya and DeLuca, 1973). The circulating concentration of 25OHD is the accepted biomarker for vitamin D status, as this reflects both dietary intake and skin production. The optimal vitamin D status has been a subject of debate and there is no general standard (Dawson-Hughes et al., 2005). Some studies indicate that vitamin D_2_ and vitamin D_3_ acts equally in maintaining vitamin D status (Rapuri et al., 2004; Holick et al., 2008), while others indicate that vitamin D_2_ is less effective than vitamin D_3_ (Trang et al., 1998; Armas et al., 2004). After production of 25OHD in the liver, it is transported, to the kidneys bound to DPB. In passing through the kidneys, 25OHD is hydroxylated at the α-position of C-1 by 1α-hydroxylase to generate 1α,25-dihydroxyvitamin D [1,25(OH)_2_D] (Jones et al., 1998; Prosser and Jones, 2004). The bioconversion of 25OHD to 1,25(OH)_2_D is strictly regulated by serum calcium and serum phosphorus levels, 1,25(OH)_2_D blood levels and parathyroid hormone (PTH) (Prosser and Jones, 2004).

### Biological effect of vitamin D

The main function of vitamin D is in maintenance and regulation of calcium and phosphorus levels in the body (DeLuca, 2004). Low blood calcium stimulates release of PTH from the parathyroid gland. In turn, PTH stimulates 1α-hydroxylase in the kidneys to produce 1,25(OH)_2_D, which then increases serum calcium and phosphorus concentrations by acting on three targets: increased absorption from the intestine, reabsorption in the kidneys and mobilization from bones (DeLuca, 2004). The active metabolite, 1,25(OH)_2_D, mediates its biological effect by binding to the vitamin D receptor (VDR). The mechanisms by which 1,25(OH)_2_D performs its biological effect can be divided into two: a genomic and a non-genomic (Norman et al., 1992). The genomic mechanism is mediated by nuclear VDRs that on binding to 1,25(OH)_2_D interacts with DNA to modulate gene expression, while the non-genomic pathway includes interactions with VDRs in the cell membrane (Norman et al., 1992). The non-genomic pathway is usually working very fast, i.e., within seconds and minutes, whereas genomic responses typically take a few hours to days (Norman, 2006).

### Dietary intake and recommended daily intake of vitamin D

Because the body produces vitamin D_3_, vitamin D does not meet the classical definition of a vitamin. Generally, fish have the highest natural amount of vitamin D_3_, e.g., salmon contains 30 μg/100 g and tuna 2.9 μg/100 g (Danish Food Composition Databank, revision. 7, 2008). Other sources of vitamin D_3_ are meat (~0.6 μg/100), egg (~1.75 μg/100) and milk products (~0.1 μg/100) (Danish Food Composition Databank, revision. 7, 2008). The content of vitamin D in food of animal origin depends on what the animal has been fed (Mattila et al., 1999; Graff et al., 2002; Jakobsen et al., 2007). The main compound in food is vitamin D_3_, but the metabolites, which are part of the metabolic pathway in vertebrates also exist (Mattila et al., 1995a,b; Clausen et al., 2003; Jakobsen and Saxholt, 2009). The potency of 25OHD has often been attributed to possess five times the potency of vitamin D (Reeve et al., 1982; Cashman et al., 2012). This value is implemented in food composition databases. However, there is no consensus on the conversion factor that should be used for 25OHD to calculate the vitamin D activity mainly because of very limited human data (Jakobsen, 2007; Cashman et al., 2012). The potency of 1,25(OH)_2_D has been attributed to ten relative to vitamin D (Tanaka et al., 1973), but this value is not implemented in food composition tables, as there is no specific composition data available for 1,25(OH)_2_D. Food sources of vitamin D_2_ are very limited and wild mushrooms are one of the only significant sources of vitamin D_2_ (Mattila et al., 1994, 2002; Teichmann et al., 2007). However, milk from dairy cows contains a significant although low amount of vitamin D_2_, which is expected to derive from grass and hay (Jakobsen and Saxholt, 2009). Vitamin D fortification of selected foods has been accepted as a strategy to improve the vitamin D status of the general population both in the United States and in many European countries. Milk and margarine are the primary products that are enriched with vitamin D (Natri et al., 2006), but also orange juice (Calvo et al., 2004), bread (Natri et al., 2006; Hohman et al., 2011), cheese and yoghurt may be enriched (Holick, 2011). This area is regulated differently in each country. Fortification may either be voluntary or mandatory and the levels added vary accordingly.

The American dietary vitamin D recommendations are 15 μg/day for the age group 1–70 years including women who are pregnant or lactating and increases to 20 μg/day for adults older than 70 years (Institute of Medicine, 2011). An adequate intake is estimated to 10 μg/day for infants (Institute of Medicine, 2011). Without sufficient vitamin D humans will develop a deficiency disease. Growing children develop rickets because of failure in calcification of cartilaginous growth plates. Osteomalacia develops in adults during prolonged vitamin D deficiency, where the newly formed uncalcified bone tissue gradually replaces the old bone tissue with weakened and soft bones as a consequence. Excessive vitamin D consumption can result in toxicity. Toxic levels are not obtained by a usual diet, but by excessive consumption of vitamin D supplements or over-fortification of food. Vitamin D intoxication is primarily due to hypercalcemia caused by increased intestinal absorption of calcium, together with increased resorption of bones. If the vitamin D exposure is prolonged, deposition of calcium in soft tissues particularly in arterial walls and in the kidney occurs. An upper intake level for vitamin D has been set to 100 μg for adults and children aged 9 years and older (Institute of Medicine, 2011).

## The discovery of vitamin D in plants

In 1924 two groups independently discovered that light exposure of inert food could result in antirachitic activity (could cure rickets) (Hess and Weinstock, 1924; Steenbock and Black, 1924). Otherwise, inert foods such as linseed oil, cottonseed oil, wheat and lettuce were made antirachitic when exposed to light from a mercury lamp (Hess and Weinstock, 1924, 1925). The question at that time was: “What was the substance in vegetables and crops that could be activated by light exposure?” Later, vitamin D_2_ was identified from solutions of irradiated ergosterol (Askew et al., 1930; Windaus, 1931). The high concentrations of ergosterol in fungi and as a result in plants contaminated with fungi led to a general perception of vitamin D_2_ as a plant form of vitamin D. Vitamin D_3_ has on the other hand been considered absent from plants. However, grazing animals in several parts of the world develop calcium intoxication, similar to that caused by vitamin D toxicity, from consuming particular plants (Mello, 2003). This is believed to be due to vitamin D_3_ or a metabolite of vitamin D_3_ present in the plants that stimulate calcium absorption producing hypercalcemia and deposition of calcium in soft tissue including aorta, heart, kidneys, intestines, and uterus (Mello, 2003). Most work has been made on the plant *Solanum glaucophyllum* Desf. (*S. glaucophyllum*) that causes calcium intoxication of livestock in South America (Mello, 2003). Controlled studies with various animals including rabbits (Mautalen, 1972; Humphreys, 1973; Dallorso et al., 2008), chickens (Wasserman et al., 1976a; Weissenberg et al., 1989) and rats (Uribe et al., 1974; Basudde and Humphreys, 1976) demonstrated that *S. glaucophyllum* leaves or extracts caused increased absorption of calcium and phosphorus similar to vitamin D. *Cestrum diurnum* L. (*C. diurnum*) and *Trisetum flavescens* Beauv. (*T. flavescens*) are also known to cause calcium intoxication very similar to *S. glaucophyllum* (Wasserman et al., 1975; Peterlik et al., 1977; Rambeck et al., 1979). Studies with these plants later led to the identification of vitamin D_3_ and related compounds in plant tissue.

## Sterols—precursors of vitamin D

**Figure 2:**
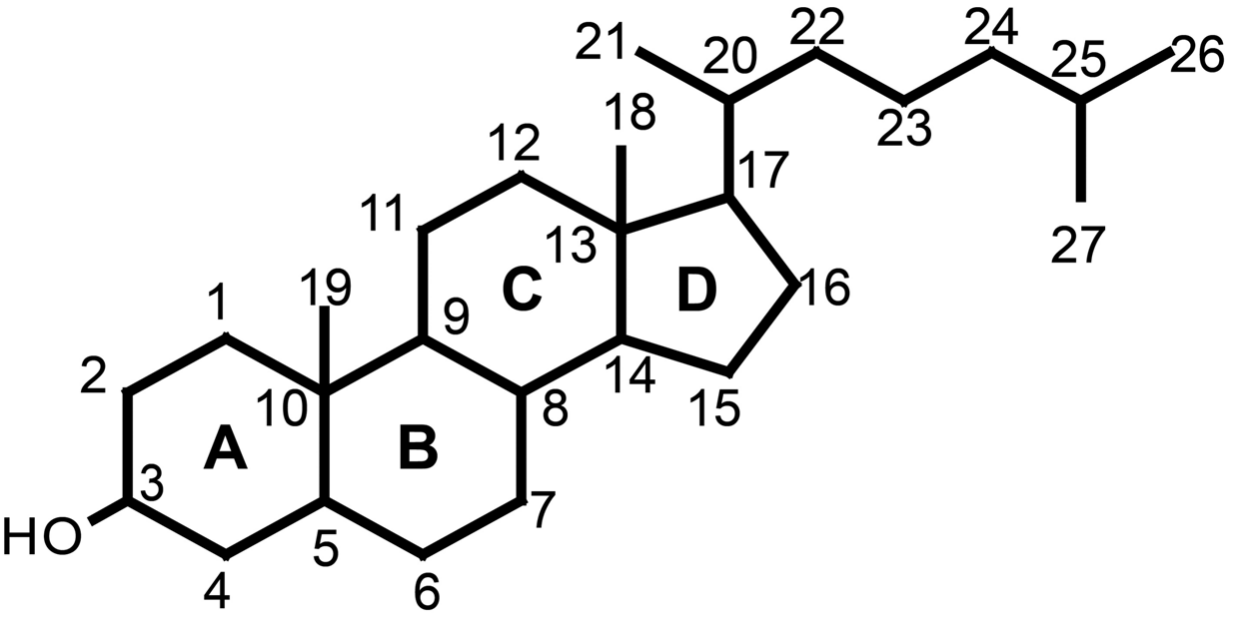
**The core structure of sterols is a fused four ring (A,B,C, and D ring).** Various groups are added to the core structure to generate a variety of sterols. Numbering of the carbon atoms is according to the 1989 IUPAC-IUB recommendations.

**Figure 3:**
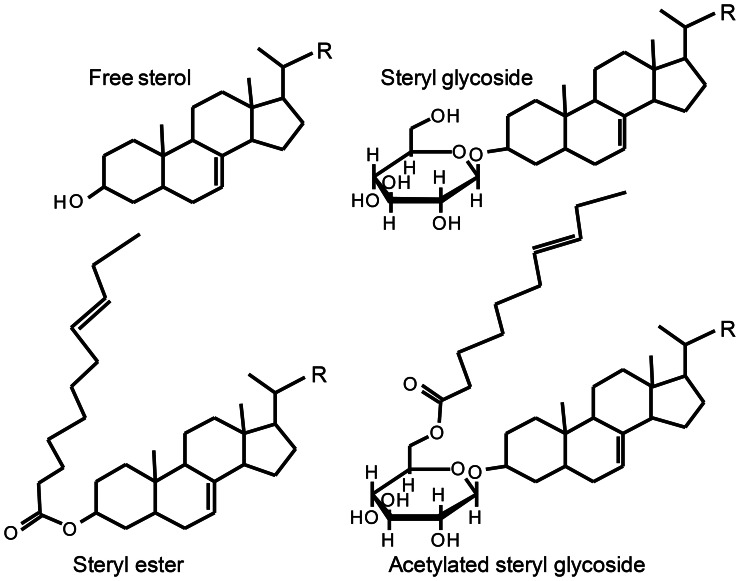
**Basic structures of free sterol and its conjugates.** The side chain R varies between various sterols. Figure Adapted from Toivo et al. (2001).

Sterols are essential for all eukaryotes. They are components of membranes and have a function in regulation of membrane fluidity and permeability (Piironen et al., 2000). Sterols also play an important role as precursors of many steroid hormones including vitamin D and brassinosteroids as well as for a wide range of secondary metabolites such as saponins and glycoalkaloids. Sterols are made up of four rings designated A, B, C, and D with one or more double bonds, a long flexible side chain at C17, a hydroxyl group attached to C3 and a variety of substituents (Figure 2). The hydroxy group at C3 can be esterified with either a long-chain fatty acid or a phenolic acid to give a steryl ester (Figure 3). Steryl esters are present in all plants, most often localized in the cytoplasm of plant cells (Benveniste, 2002), and represent a storage form of sterols (Piironen et al., 2000). The 3-hydroxy group can also be linked to a carbohydrate forming a steryl glycoside (Figure 3). Steryl glycosides usually consist of a mixture differing in carbohydrate moiety and esterification of the sugar by a fatty acid can give rise to an acetylated steryl glycoside (Figure 3). Especially, plants from the *Solanaceae* family show a unique abundance of glycosides (Moreau et al., 2002; Potocka and Zimowski, 2008).

Generally, sterols can be divided into three groups according to the alkylations at the C24 position in the side chain: 24-desmethylsterols (without an alkyl group), 24-demethylsterols (with one methyl group) and 24-ethylsterols (with one ethyl group). 24-desmethylsterols are typical for animals, whereas the 24-demethylsterols and 24-ethylsterols are typical for plants and fungi. Animals and fungi accumulate the major end product sterols, cholesterol (24-desmethylsterol) and ergosterol (24-demethylsterol), whereas the plant kingdom in comparison produces a wide range of sterols. More than 250 sterols have been found in plants (Hartmann, 2004), but sitosterol, campesterol, and stigmasterol normally predominates (Lagarda et al., 2006). Plant sterols typically have a double bond between C5 and C6 in the B ring and are called Δ5-sterols. Sterols with a Δ5 nucleus are the most common, but Δ7-sterols, Δ5,7-sterols, and Δ22-sterols also occur (Piironen et al., 2000). Plant tissues contain an average quantity of 1–3 mg sterols per gram dry weight (Schaller, 2004). The sterol composition of plant species is genetically determined and varies considerably (Schaller, 2003). The model plant, *Arabidopsis thaliana*, e.g., has a sterol composition of 64% sitosterol, 11% campesterol, 6% stigmasterol, 3% isofucosterol, 2% brassicasterol and 14% of other minor sterols (Schaeffer et al., 2001). Cholesterol is the major sterol in animals, but is also present in plants. Usually, cholesterol accounts for 1–2% of total plant sterols, but higher levels are present in especially *Solanaceae* (Whitaker, 1988, 1991; Z1993adlo, 1993; Moreau et al., 2002; Jäpelt et al., 2011b). It has been suggested that cholesterol serves as a precursor of glycoalkaloids (Bergenstråhle et al., 1996) and ecdysteroids (Dinan, 2001) in these plants.

## Vitamin D biosynthesis

**Figure 4:**
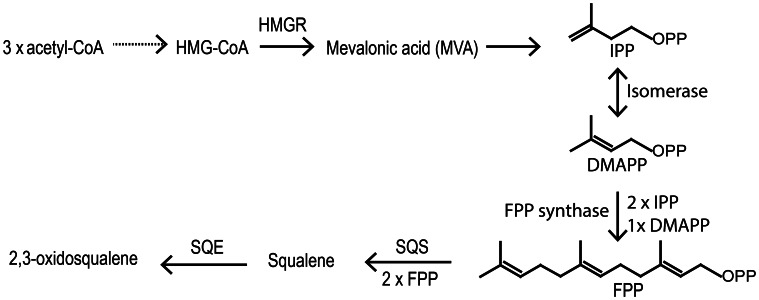
**First part of the biosynthetic pathway of sterols.** Solid arrows mean one reaction and doted arrow multiple steps. HMG-CoA, 3-hydroxymethyl-3-glutaryl coenzyme A; HMGR, HMG-CoA reductase; IPP, Isopentyl pyrophosphate; DMAPP, Dimethylalkyl pyrophosphate; FPP, Farnesyl pyrophosphate; SQS, squalene synthase; SQE, squalene epoxidase.

**Figure 5:**
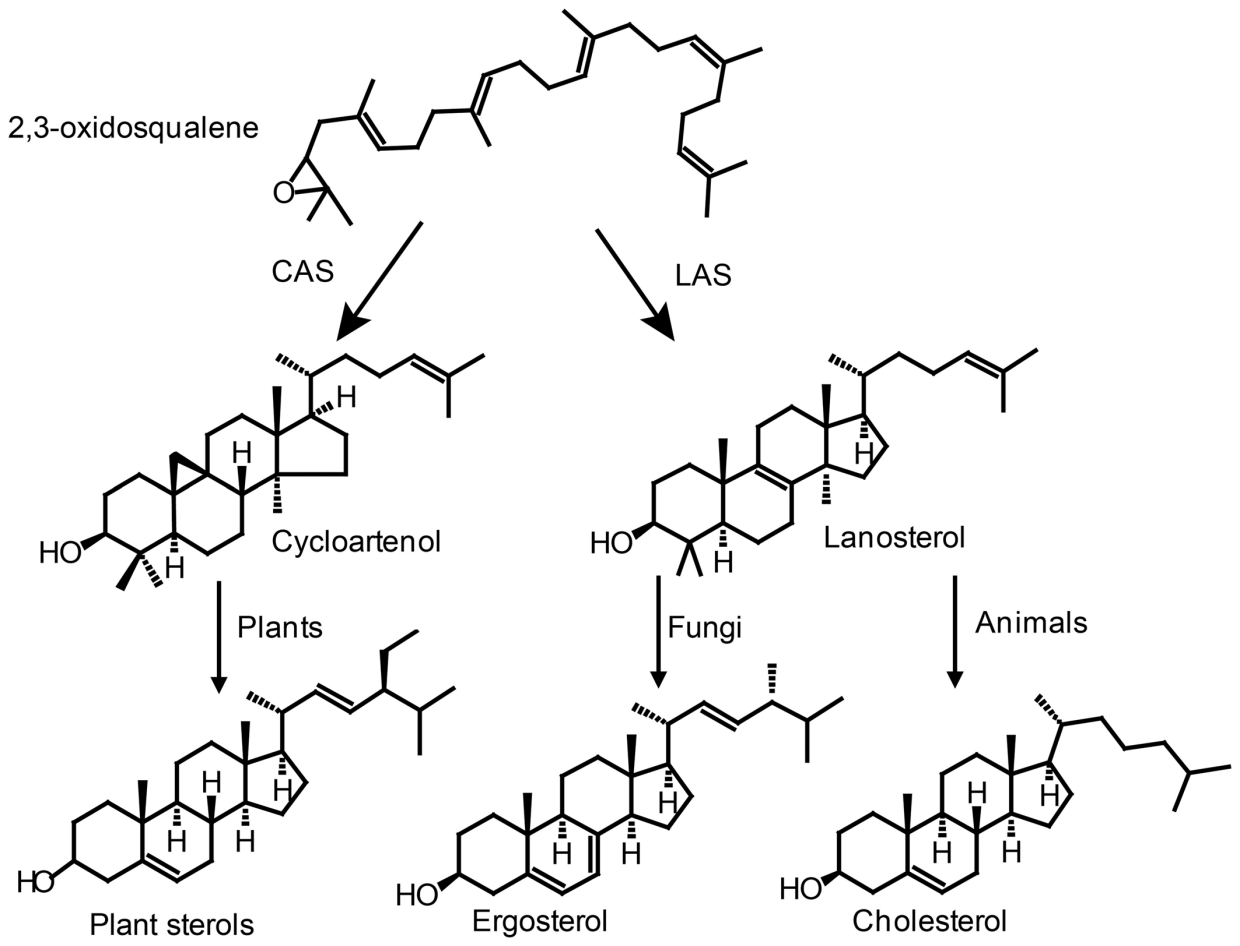
**Cyclization of 2,3-oxidosqualene forms either lanosterol or cycloartenol via a series of enzymatic cyclizations leading to sterols in plants, fungi and animals.** CAS, cycloartenol synthase; LAS, lanosterol synthase.

Vitamin D biosynthesis is taking place along the normal sterol pathway, i.e., vitamin D_2_ is formed by UVB exposure of ergosterol and vitamin D_3_ by UVB exposure of 7-dehydrocholesterol. Therefore, we need to understand how its sterol precursors are formed in order to understand how vitamin D synthesis takes place in plants. Sterol biosynthesis can be divided into two parts. The first part is the mevalonic acid pathway. All isoprenoid compounds, including sterols, are formed via the mevalonic acid pathway from the common C5 isoprene building blocks isopentyl diphosphate (IPP) and its isomer dimethylallyl diphosphate (DMAPP) (Figure 4). One molecule DMAPP and two molecules IPP is assembled to farnesyl pyrophosphate (FPP) (Figure 4). Finally, two molecules FPP are combined to make squalene (Hartmann, 2004). Cyclization of squalene is via the intermediate 2,3-oxidosqualene, that forms either lanosterol or cycloartenol via a series of enzymatic cyclizations (Figures 4, 5). Animals and fungi forms lanosterol catalyzed by lanosterol synthase (LAS) and plants form cycloartenol catalyzed by cycloartenol synthase (CAS) (Figure 5). Several reviews have covered the enzymes and genes involved in the sterol pathway (Benveniste, 1986, 2002, 2004; Bach and Benveniste, 1997; Schaller, 2003, 2004; Hartmann, 2004; Nes, 2011). Therefore, only the steps downstream from 2,3-oxidosqualene relevant for the biosynthesis of vitamin D_2_ and vitamin D_3_ will be included in the present review.

### Sterol biosynthesis leading to vitamin D_3_—animals

**Figure 6:**
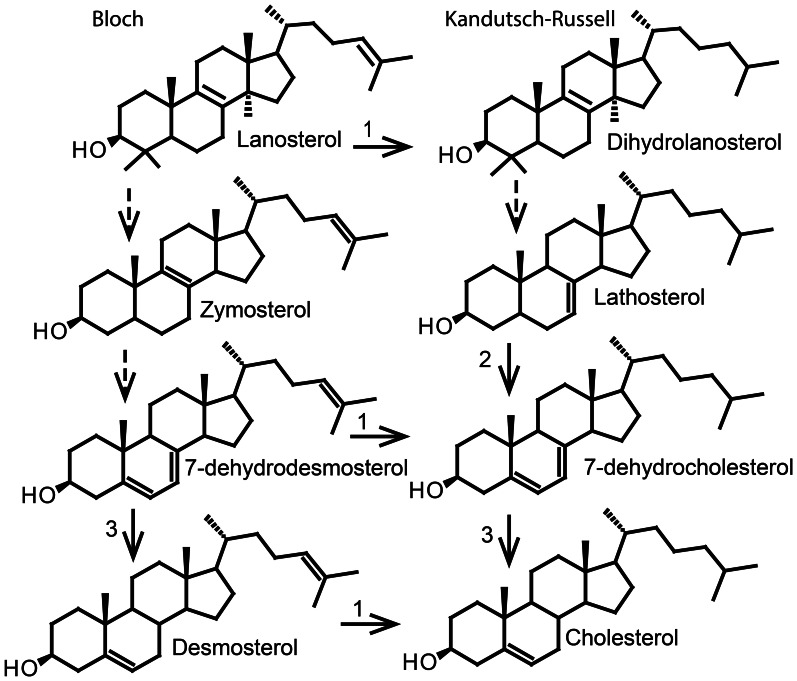
**Simplified cholesterol biosynthesis.** Lanosterol is converted to cholesterol in a series of enzyme reactions. Dashed arrows indicate more than one biosynthetic step. Solid arrows indicate a biosynthetic step regulated by: (1), sterol-Δ^24^-reductase; (2), lathosterol 5-desaturase; (3), 7-dehydrocholesterol reductase.

The major end product of the animal sterol pathway is cholesterol synthesized via lanosterol (Figure 5). The conversion of lanosterol to cholesterol requires nine different enzymes (Risley, 2002; Nes, 2011) and involves removal of three methyl groups, reduction of double bonds and migration of a double bond in lanosterol to a new position in cholesterol (Waterham et al., 2001). Two intersecting routes to cholesterol have been postulated (Nes, 2011). The direction of the pathway is determined by the stage at which the double bond at C24 in the sterol side chain is reduced (Nes, 2011). In the Kandutsch–Russell pathway, the reduction of the C24 double bond happens as the first step (Kandutsch and Russell, 1960). The final precursor for cholesterol in the Kandutsch–Russell pathway is 7-dehydrocholesterol and the last step a reduction of the Δ7 double bond by a Δ^5, 7^-sterol-Δ^7^-reductase (7-dehydrocholesterol reductase) to give cholesterol (Figure 6). Desmosterol is the ultimate precursor of cholesterol in the Bloch pathway (Bloch, 1983) (Figure 6). Desmosterol is converted to cholesterol in the final step of the pathway by a sterol-Δ^24^-reductase. However, the sequence of reactions in the cholesterol biosynthetic pathway may vary (Waterham et al., 2001). Alternate routes exist because reduction of the C24–C25 double bond in the side chain by sterol Δ^24^-reductase can occur on all intermediates between lanosterol and desmosterol in the Bloch Pathway, giving rise to various intermediates (Bae and Paik, 1997). These intermediates can serve as substrates in the Kandutsch-Russell pathway as shown for 7-dehydrodesmosterol in Figure 6.

### Sterol biosynthesis leading to vitamin D_2_—fungi

**Figure 7:**
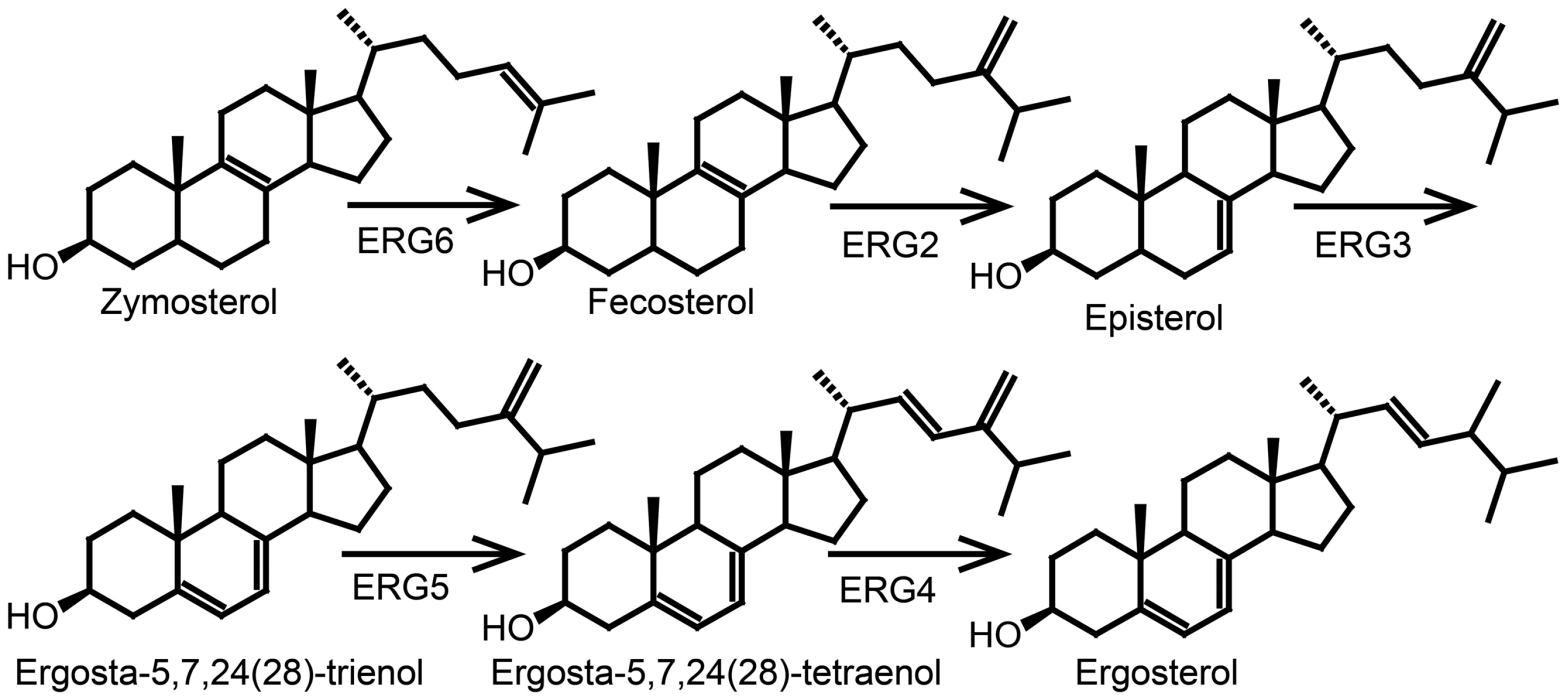
**The last five steps of the ergosterol biosynthetic pathway in *Saccharomyces cerevis*.** ERG6, *S*-adenosylmethionine sterol methyltransferase; ERG2, C8-C7 sterol isomerase; ERG3, Δ^5^-desaturase; ERG5, Δ^22^-desaturase; ERG4, Δ^24^-reductase. Figure adapted from Lees et al. (1995).

The major sterol end product in fungi is ergosterol synthesized via lanosterol (Figure 5). The yeast *Saccharomyces cerevisiae* has been used as a model system for the elucidation of the ergosterol pathway and all enzymes involved have been identified (Lees et al., 1995). Cholesterol and ergosterol share the pathway until zymosterol (Figure 6) (Lees et al., 1995). However, sterols from fungi differ from animal sterols by the presence of a methyl group at C24. The alkylation of the side chain is catalyzed by *S*-adenosylmethionine sterol methyltransferase (ERG6) that in *S. cerevisiae* converts zymosterol into fecosterol (Figure 7) (Bach and Benveniste, 1997). Plants are not known to produce ergosterol, and any vitamin D_2_ present is probably derived from endophytic fungi or a fungal infection.

### Sterol biosynthesis leading to vitamin D_3_—plants

**Figure 8:**
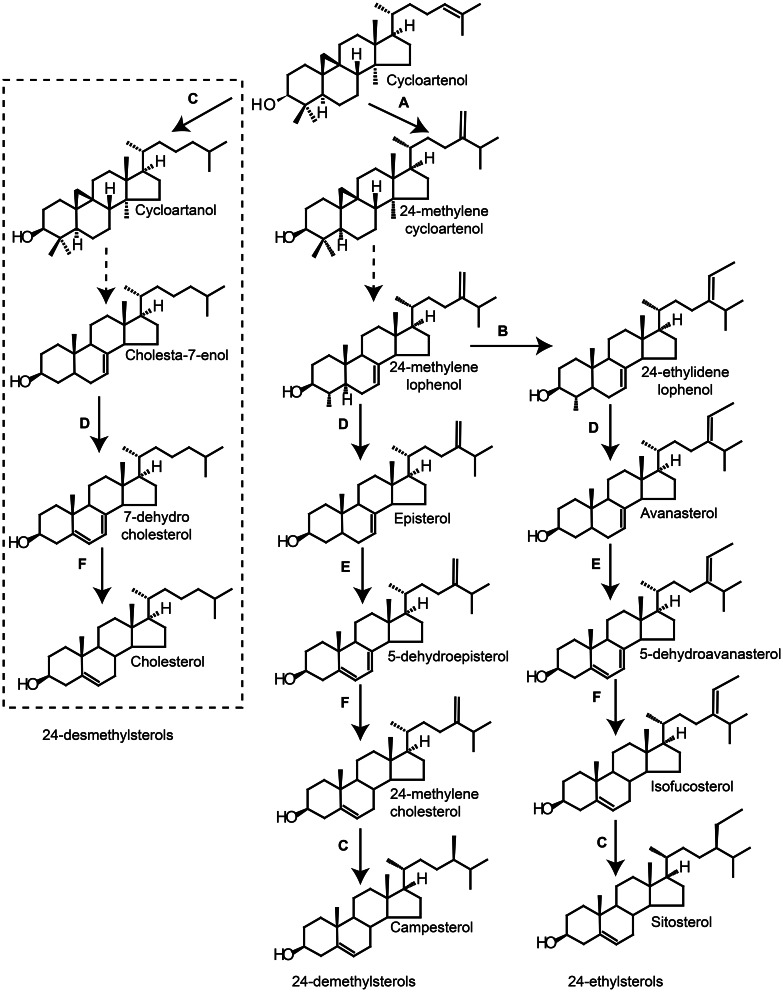
**The figure represents the biosynthetic pathways to sterols downstream from cycloartenol.** Hypothetical pathway for 24-desmethylsterols (left) marked with dashed box. Simplified biosynthetic pathways for 24-demethylsterols (middle) and 24-ethylsterols (right). Dashed arrows indicate more than one biosynthetic step. Solid arrows indicate a biosynthetic step regulated by: **(A)** SMT1, **(B)** SMT2, **(C)** DIM/DWARF1, **(D)** DWARF7/STE1, **(E)** C4-demethylase and **(F)** DWARF5.

The enzymes involved in 24-demethylsterol and 24-ethylsterol synthesis have been identified in the model plant *Arabidopsis thaliana*. However, the biosynthetic pathway for 24-desmethylsterols, such as cholesterol and 7-dehydrocholesterol, remains unknown. This is probably due to the fact that these are minor sterols in Arabidopsis as well as in most other plants. Experiments with biosynthetic mutants and transgenic plants indicate that the enzymes regulating 24-demethylsterols and 24-ethylsterols also are involved in the regulation of 24-desmethylsterols. Within this chapter a hypothetical biosynthetic route to 24-desmethylsterols with cholesterol as end product will be presented (Figure 8).

#### Lanosterol as an alternative precursor for sterols

Plant sterols are synthesized via cycloartenol catalyzed by CAS (Figure 5). However, evidence exists of the presence of putative LAS genes in *Arabidopsis thaliana* (Kolesnikova et al., 2006; Suzuki et al., 2006; Ohyama et al., 2009), *Panax Ginseng* (Suzuki et al., 2006) and *Lotus japonica* (Kolesnikova et al., 2006; Sawai et al., 2006). Consequently, lanosterol synthesized by LAS in plants may act as an alternative intermediate for sterol synthesis. A lanosterol pathway to plant sterols has been demonstrated in Arabidopsis (Ohyama et al., 2009). The lanosterol pathway only contributed to 1.5% of the sitosterol biosynthesis, but this was increased to 4.5% by LAS overexpression (Ohyama et al., 2009). Thus, sterols in plants may be synthesized by two biosynthetic routes, via cycloartenol and/or via lanosterol. As a result cholesterol and 7-dehydrocholesterol may be formed in plants through lanosterol as is known from animals (Figure 6). In future experiments, it has to be confirmed if plants producing high amounts of these sterols such as *Solanaceae*, have a more efficient LAS enzyme.

#### S-adenosylmethionine sterol methyltransferases (SMTs)

Sterols from plants differ from animal sterols by the presence of a methyl or an ethyl group at C24. *S*-adenosylmethionine sterol methyltransferases (SMTs) catalyze the transfer of two carbon atoms from *S*-adenosyl methionine to make the 24-alkylations and are considered important regulatory steps in the biosynthesis of sterols in plants (Schaller, 2003). The alkyl substituent at C24 is the product of either one single carbon addition or two single carbon additions. The two methyl additions are performed as distinct steps in the pathway and two classes of SMTs exist: SMT1 and SMT2 (Figure 8). SMT1 preferably catalyze the first methylation of cycloartenol to give 24-methylenecycloartenol (Hartmann, 2004) (Figure 8). The ratio of cholesterol and the major plant sterols sitosterol, stigmasterol and campesterol has been shown to be controlled by the activity of SMT1 (Hartmann, 2004). In *Arabidopsis thaliana* plants, bearing a SMT1 knockout, cholesterol was the major sterol, composing 26% of total sterols, compared with 6% in wild-type plants (Diener et al., 2000). The *smt*1 mutant displayed poor growth and fertility, root sensitivity to Ca^2+^ and loss of proper embryo morphogenesis (Diener et al., 2000). SMT1 overexpressing tobacco plants do in contrast have a reduced content of cholesterol and no visual phenotype (Schaeffer et al., 2000; Sitbon and Jonsson, 2001; Holmberg et al., 2002). Similar results have been observed in transgenic potato (*Solanum tuberosum* cv Désirée) overexpressing SMT1 (Arnqvist et al., 2003). These results indicate that the production of high amounts of cholesterol in plants results from a by-pass of SMT1. Thus, manipulation of SMT1 might be a tool to increase the 7-dehydrocholesterol and cholesterol content in plants.

#### Proposed steps of the 24-desmethylsterol biosynthesis

Several enzymes involved in the sterol biosynthesis can be found across plants and animals, e.g., Δ^5, 7^-sterol-Δ^7^-reductase called DWARF5 in plants and 7-dehydrocholesterol reductase in animals. Several of these enzymes do not have absolute substrate specificity (Benveniste, 1986). The possibility, therefore, exist that plant biosynthetic enzymes could be involved in 24-desmethyl sterol biosynthesis. Application of cycloartanol to growing tobacco plants generates cholesterol (Devys et al., 1969) and we hypothesize that the reduction of the Δ24 double bond of cycloartenol to yield cycloartanol is the first step of cholesterol synthesis in plants (Figure 8). In Arabidopsis, the Δ24-reduction step is catalyzed by Δ5-sterol-Δ24-reductase (DIM/DWARF1) (Klahre et al., 1998). Interestingly, the Arabidopsis *dim* mutant (Klahre et al., 1998) and also the rice *dim* mutant has decreased levels of cholesterol compared to the wild type (Hong et al., 2005). These results indicate a role of DIM/DWARF1 in cholesterol biosynthesis. Production of 7-dehydrocholesterol in animals involves a Δ^7^-sterol-C-5-desaturase (lathosterol 5-desaturase) that introduces a double bond at C5 (Figure 6). A similar Δ^7^-sterol-C-5-desaturase (DWARF7/STE1) exists in plants, which converts episterol/avanesterol into 5-dehydroepisterol/5-dehydroavanesterol by a removal of two protons (Figure 8). An Arabidopsis mutant (*ste1/dwarf7*) defective in the Δ^7^-sterol-C-5-desaturase has been identified, which only produces limiting amounts of Δ^5, 7^-sterols (Gachotte et al., 1995, 2002; Choe et al., 1999; Husselstein et al., 1999). It can be hypothesized that a *ste1/dwarf7* mutant would be defective in converting cholesta-7-enol to 7-dehydrocholesterol and further to cholesterol (Figure 8). However, no significant decrease in cholesterol levels was observed in *ste1* mutants (Gachotte et al., 1995; Husselstein et al., 1999). The enzymatic step after C5 reduction is mediated by a Δ^5, 7^-sterol-Δ^7^-reductase called DWARF5 in plants (Figure 8) and 7-dehydrocholesterol reductase in animals (Figure 6). DWARF5 e.g., catalyze the reduction of the Δ7 double bond in 5-dehydroepisterol to give the Δ5 sterol 24-methylenecholesterol (Choe et al., 2000). We propose that DWARF5 also act on 7-dehydrocholesterol to form cholesterol in plants (Figure 8). An Arabidopsis *dwarf5* mutant accumulating Δ^5, 7^-sterols has been identified (Choe et al., 2000). The DWARF5 mutant display a characteristic dwarf phenotype, which includes short robust stems, reduced fertility, prolonged life cycle and dark-green curled leaves when grown in light (Choe et al., 1999). The special dwarf phenotype is explained by a deficiency in brassinosteroids, which are important growth hormones for plants (Klahre et al., 1998; Choe et al., 1999, 2000; Hong et al., 2005). It is possible that vitamin D_3_ producing plants have a less efficient DWARF5 enzyme that allows for accumulation of 7-dehydrocholesterol and later vitamin D_3_ by photoconversion.

## Occurrence of vitamin D_3_ and its metabolites in plants

### Provitamin D_3_ and vitamin D_3_

Vitamin D_3_ and its provitamin 7-dehydrocholesterol have been identified in the leaves of several plant species mostly belonging to *Solanaceae* (Esparza et al., 1982; Prema and Raghuramulu, 1994, 1996; Aburjai et al., 1998; Curino et al., 1998; Skliar et al., 2000) (Table 1). Huge variations exist in the content of vitamin D_3_ and 7-dehydrocholesterol (Table 1). Some studies used plant cell cultures instead of whole plants (Aburjai et al., 1996; Curino et al., 1998, 2001; Skliar et al., 2000), which may explain some of the variability between studies. Growth conditions are easily controlled when using plant cells, but discrepancies between *in vitro* and *in vivo* can be seen due to transformations occurring in the culture medium (Curino et al., 2001). Differences in growth conditions, e.g., the intensity of the light source and length of exposure will have a significant impact on the vitamin D_3_ content, but unfortunately growth conditions are poorly described in most studies (Prema and Raghuramulu, 1994, 1996; Aburjai et al., 1998). However, vitamin D_3_ has been studied in *S. lycopersicum* grown in greenhouse with or without UVB exposure (Björn and Wang, 2001) and in *S. lycopersicum*, *S. glaucophyllum* and *C. annuum* using growth chambers, an UVB lamp and controlled temperature and light/day settings (Jäpelt et al., 2011b, 2012). Vitamin D_3_ has in most studies been identified after UVB exposure (Zucker et al., 1980; Aburjai et al., 1996; Björn and Wang, 2001; Jäpelt et al., 2011b), but vitamin D_3_ synthesis without the action of UVB has also been reported (Curino et al., 1998; Jäpelt et al., 2012). Recently, we compared vitamin D_3_ in UVB- and non-UVB-exposed plants using a sensitive liquid chromatography electrospray ionization tandem mass spectrometry (LC-ESI-MS/MS) method. The content of vitamin D_3_ in the UVB-exposed plants was 18–64 times higher than for the non-UVB-exposed plants (Jäpelt et al., 2012). Previously failure to detect vitamin D_3_ in non-UVB-exposed plants could be due to the use of relative insensitive analytical methods. Since the isomerization of previtamin D_3_ to vitamin D_3_ is a temperature-dependent reaction an effect of growth temperature could be expected. Therefore, the effect of elevated temperature and a combination of elevated temperature and UVB light were investigated in *S. lycopersicum*, *S. glaucophyllum* and *C. annuum* (Jäpelt et al., 2012). Plants were kept in a growth chambers for 7 days at 32°C either exposed to UVB light or not, but no consistent effect was seen. In *S. glaucophyllum* the value for elevated temperatures combined with UVB was half the value for UVB alone, whereas in *S. lycopersicum* and *C. annuum* a small increase for elevated temperatures combined with UVB compared to UVB alone was observed (Jäpelt et al., 2012).

**Table 1:** **Content of vitamin D_3_ and provitamin D_3_ (μg/g) in various plants determined with chemical methods**.

**Species**	**Reference no.**	**Vitamin D_3_**	**Provitamin D_3_**	**Method**
*Solanum lycopersicum*	1	0.28 μg/g dry wt.	0.61–0.76 μg/g dry wt.	Not stated
	2	0.09 μg/g dry wt.	0.23–0.47 μg/g dry wt.	LC-APCI-MS/MS
	3	1.1 μ g/g fresh wt.	n.a	HPLC-UV and identification by H NMR and MS
	4	0.8 μg/g dry wt.	n.a.	HPLC-UV
	5	0.1 μg/g dry wt. (+UVB) 0.002 μg/g dry wt. (−UVB)		LC-ESI-MS/MS with derivatization
*Solanum tuberosum*	3	0.15 μ g/g fresh wt.	n.a	HPLC-UV and identification by H NMR and MS
*Cucurbita pepo*	3	0.23 μ g/g fresh wt.	n.a.	HPLC-UV and identification by H NMR and MS
*Solanum glaucophyllum*	2	0.21 μg/g dry wt.	0.67–1.26 μg/g dry wt.	LC-APCI-MS/MS
	5	0.1–0.2 μg/g dry wt. (+UVB) 0.0032-0.0055 μg/g dry wt. (−UVB)	n.a.	LC-ESI-MS/MS with derivatization
	6	2.2–42.1μ g/g fresh wt.	5–58μ g/g fresh wt.	HPLC-UV and identification by H NMR and MS
	7,8	ID	ID	HPLC-UV and identification by MS
*Nicotiana glauca*	9	ID	ID	HPLC-UV and identification by MS
*Cestrum diurnum*	10	0.1 μ g/g dry wt.	n.a.	HPLC-UV
*Medicago sativa*	11	0.00062-0.001 μg/g dry wt.	n.a.	HPLC-UV with identification by MS
*Trisetum flavescens*	12	0.1 μg/g dry wt.	n.a.	GC-MS
*Capsicum annuum*	2	<LOD	0.03μg/g	LC-APCI-MS/MS
	5	0.0029–0.0063 μg/g dry wt (+UVB)	n.a.	LC-ESI-MS/MS with derivatization

n.a., not analysed; ID, identified not quantified; <LOD, below detection limit.

1, (Björn and Wang, 2001); 2, (Jäpelt et al., 2011b); 3, (Aburjai et al., 1998); 4, (Prema and Raghuramulu, 1996); 5, (Jäpelt et al., 2012); 6, (Aburjai et al., 1996); 7, (Curino et al., 2001); 8, (Curino et al., 1998); 9, (Skliar et al., 2000); 10, (Prema and Raghuramulu, 1994); 11, (Horst et al., 1984); 12, (Rambeck et al., 1979).

### Hydroxylated metabolites of vitamin D_3_

Hydroxylated metabolites of vitamin D_3_ have been found in various plants (Table 2). The highest content of 1,25(OH)_2_D_3_ has been found in *S. glaucophyllum* and not only in the leaves, but also in fruits, stems and roots (Weissenberg et al., 1989; Curino et al., 2001). The level of 1,25(OH)_2_D_3_ found in cell cultures varied according to the origin of the culture, i.e., stem > leaf > fruit (Curino et al., 2001). However, vitamin D like activity could not been found in tomatoes (Prema and Raghuramulu, 1996). Earlier work using cell cultures indicates that the production of hydroxylated metabolites is influenced by the calcium concentration (Aburjai et al., 1997; Curino et al., 2001; Burlini et al., 2002). The level of 25OHD_3_ (Aburjai et al., 1997) and 1,25(OH)_2_D_3_ (Burlini et al., 2002) in *S. glaucophyllum* cell suspensions increased markedly when incubated in a Ca^2+^ free media compared to if Ca^2+^ was present. However, another study performed with *S. glaucophyllum* cells showed that media deprived from calcium contained low levels of 1,25(OH)_2_D_3_ (Curino et al., 2001).

**Table 2:** **Content of the hydroxylated metabolites of vitamin D_3_ in various plants determined by immunoassays or chemical methods**.

**Species**	**Reference no.**	**25OHD_3_**	**Method**	**1,25(OH)_2_D_3_**	**Method**
*Solanum lycopersicum*	1	0.15 μ g/g fresh wt.	HPLC-UV and MS identification	<LOD	HPLC-UV
	2	0.022 μg/g dry wt.	HPLC-UV and biological activity	0.10 μg/g dry wt.	HPLC-UV and biological activity
	3	0.004 μg/g dry wt	LC-ESI-MS/MS with derivatization	<LOD	LC-ESI-MS/MS with derivatization
*Solanum glaucophyllum*	3	0.011–0.031 μg/g dry wt	LC-ESI-MS/MS with derivatization	0.012–0.032 μg/g dry wt	LC-ESI-MS/MS with derivatization
	4	ID	HPLC-UV and MS identification	ID	Radioreceptor assay, HPLC- UV and MS identification
	5	1.0 μg/g fresh wt.	HPLC-UV and MS and HNMR identification	0.1 μg/g fresh wt.	HPLC-UV and MS and HNMR identification
*Capsicum annuum*	3	0.0004–0.0005 μg/g dry wt	LC-ESI-MS/MS with derivatization	<LOD	LC-ESI-MS/MS with derivatization
*Cestrum diurnum*	6	0.102 μg/g dry wt.	HPLC-UV and biological activity	1 μg/g dry wt.	HPLC-UV and biological activity
*Nicotiana glauca*	7	ID	HPLC-UV with MS identification	0.3–1 μg/g fresh wt.	Radioreceptor assay, HPLC- UV and MS identification

ID, identified not quantified; <LOD, below detection limit.

1, (Aburjai et al., 1998); 2, (Prema and Raghuramulu, 1996); 3, (Jäpelt et al., 2012); 4, (Curino et al., 1998); 5, (Aburjai et al., 1996); 6, (Prema and Raghuramulu, 1994); 7, (Skliar et al., 2000).

The biosynthesis of 1,25(OH)_2_D_3_ is finely regulated in vertebrates and the question is if this also is the case in plants. The 25OHD_3_/1,25(OH)_2_D_3_ ratio has either been reported to be >1 (Prema and Raghuramulu, 1994, 1996), ~1 (Jäpelt et al., 2012) or <1 (Aburjai et al., 1996). This indicates that the conversion of 25OHD_3_ to 1,25(OH)_2_D_3_ not is as tightly regulated as in vertebrates. Enzymatic activities involved in formation of 25OHD_3_ and 1,25(OH)_2_D_3_ have been identified in *S. glaucophyllum* (Esparza et al., 1982). Vitamin D 25-hydroxylase activity has been localized in the microsomes, whereas the 1α-hydroxylase activity has been localized in mitochondria and microsomes (Esparza et al., 1982). However, no enzymes have been isolated from plants showing vitamin D 25-hydroxylase or 1α-hydroxylase catalytic activity. For the biological role of vitamin D_3_ and its hydroxylated metabolites in plant physiology is referred to Boland et al. (2003).

### Vitamin D_3_ conjugates

Several studies of *S. glaucophyllum* identified 1,25(OH)_2_D_3_ after enzymatic hydrolysis with glycosidases (Haussler et al., 1976; Wasserman et al., 1976b; Hughes et al., 1977; Napoli et al., 1977; Esparza et al., 1982; Jäpelt et al., 2012) and also in *C. diurnum* (Hughes et al., 1977). Similarly, vitamin D_3_ and 25OHD_3_ were identified in *S. glaucophyllum* after incubation with glycosidases (Esparza et al., 1982). Ruminal fluids contain glycosidases and research show that aqueous extracts of *S. glaucophyllum* leaves incubated with bovine ruminal fluid (de Boland et al., 1978) and ovine ruminal fluid (Esparza et al., 1983) exhibit more vitamin D activity than extracts not incubated. Later vitamin D_3_ and its metabolites were identified in *S. glaucophyllum* extracts incubated with ovine ruminal fluid (Skliar et al., 1992). These studies indicate that vitamin D_3_ and its metabolites exist as glycosides in plants. However, the existence of glycosides is debated and other studies only quantified the free forms (Prema and Raghuramulu, 1994, 1996; Aburjai et al., 1996, 1997, 1998). It has been proposed that the glycoside content depends on the collection, drying and storage of the plant material, which may explain some differences between studies (Peterlik et al., 1977; Prema and Raghuramulu, 1994).

The site of glycosylation, the type of bond and the identity of the sugar unit have not been completely determined. The number of sugar units seems to differ as the vitamin D active glycosides of *S. glaucophyllum* and *T. flavescens* are soluble in water (Humphreys, 1973; Uribe et al., 1974; Wasserman et al., 1976b; Napoli et al., 1977; Morris and Levack, 1982), whereas the glycoside of *C. diurnum* are soluble in a mixture of chloroform and methanol (Wasserman et al., 1976a). Vidal et al. (1985) isolated the 1,25(OH)_2_D_3_ glycoside from *S. glaucophyllum* and found that 1,25(OH)_2_D_3_ was bound to a series of fructoglucosides of variable molecular weights.

The formation of glycosides may cause dramatic changes in the chemical, nutritional and metabolic properties (Gregory, 1998). Rambeck et al. (1984) studied the biological activity of 1α(OH)D_3_ 3-β-cellobioside, 1α(OH)D_3_ 3-β-glucoside and vitamin D_3_ 3-β-glucoside and the corresponding parent molecules in bioassays using rats, chickens and quails. Glucosidation did not reduce the activity of the parent vitamin D (Rambeck et al., 1984). In constrast the β-D-glucoside of 1α(OH)D_3_ exhibited only 10% activity compared to 1α (OH)_2_D_3_ in all bioassays and the disaccharide (1α (OH)vitamin D_3_ 3-β-cellobioside) showed no vitamin D activity in the chicken bioassay (Rambeck et al., 1984). No study on glycosylated forms of 25OHD_3_ or 1,25(OH)_2_D_3_ has been performed. The existence of esters of vitamin D and the hydroxylated metabolites in plant material seems likely, but has not been shown.

## Vitamin D_2_ in plant material

Ergosterol is a cell membrane component of fungi, but is also the provitamin of vitamin D_2_. Thus, vitamin D_2_ can be found in plants contaminated with fungi. Conversion to vitamin D_2_ occurs by sun-exposure of the plant material during growth and in the curing process. The antirachitic activity of grass and hay was studied intensively 50–80 years back using rat assays (Steenbock et al., 1925; Russell, 1929; Wallis, 1938, 1939; Moore et al., 1948; Newlander, 1948; Thomas and Moore, 1951; Newlander and Riddell, 1952; Thomas, 1952; Keener, 1954; Henry et al., 1958; Wallis et al., 1958). Most of these studies were on alfalfa (*Medicago sativa* L.) and the activities ranged from 0–3800 IU/kg, equivalent to 0–95 μg vitamin D/kg. However, some studies were on hay and others on fresh grass and dry matter was not stated in all cases, which makes comparisons difficult. The assumption at that time was that the antirachitic activity was due to vitamin D_2_ produced from ergosterol (Newlander and Riddell, 1952). Later vitamin D_2_ was identified in crops using chemical methods (Horst et al., 1984; Jäpelt et al., 2011a). Horst et al. (1984) analyzed sun-cured field grown alfalfa using high performance liquid chromatography (HPLC) with UV detection and found 48 μg vitamin D_2_/kg. Jäpelt et al. (2011a) studied ergosterol and vitamin D_2_ in six varieties of perennial ryegrass (*Lolium perenne* L.) harvested four times during the season. The content of vitamin D_2_ and ergosterol was analyzed by LC atmospheric pressure chemical ionization tandem mass spectrometry (LC-APCI-MS/MS). An average content of vitamin D_2_ of 2 μg/kg fresh weight (0.07–6.4 μg/kg fresh weight) was found (Jäpelt et al., 2011a). The vitamin D_2_ content was maximum 2‰ of the ergosterol content (Jäpelt et al., 2011a). The vitamin D_2_ content in these two studies is almost similar if we take into account the difference in dry matter between hay and fresh grass. Compared to results obtained by rat assays, the latter is slightly higher, which could be due the contribution of other vitamin D metabolites, or to a natural decline during the last 50–80 years.

The content of vitamin D_2_ in the plant material has been shown to increase with the level of sun exposure, and for crops also curing method (Hess and Weinstock, 1924; Steenbock et al., 1925; Russell, 1929; Newlander and Riddell, 1952). However, inconsistent results were obtained regarding the importance of sun exposure, which indicates that other factors may be important (Moore et al., 1948; Newlander, 1948; Henry et al., 1958; Wallis et al., 1958). Several studies observed that plants at later stage of maturity were higher in vitamin D than at early stage (Thomas and Moore, 1951; Keener, 1954; Henry et al., 1958). Especially, dead leaves were high in vitamin D and the proportion of dead leaves was observed to increase with maturity of the plant (Thomas and Moore, 1951). Newell et al. (1996) measured the ergosterol content in grass and found that it increased with time and with increasing fungal damage. Consequently, larger vitamin D activities are observed with time if the plant is exposed to sunlight. Recently, a systematic study looking at vitamin D_2_ and ergosterol in perennial ryegrass during the season was performed (Jäpelt et al., 2011a). The content of both ergosterol and vitamin D_2_ changed more than a factor of 10 during the season (Jäpelt et al., 2011a). Weather factors were recorded and a principal component analysis (PCA) was performed to study, which factors that were important for the formation of vitamin D_2_. The PCA revealed that both sun/temperature and ergosterol/precipitation was important. This suggested that a combination of weather factors was involved as observed previously (Moore et al., 1948; Newlander, 1948; Henry et al., 1958; Wallis et al., 1958). Precipitation and high humidity are essential for ergosterol synthesis, whereas sunlight is necessary for vitamin D_2_ synthesis (Jäpelt et al., 2011a).

## Vitamin D in algae

Fish are known to be rich sources of vitamin D_3_, but the origin of vitamin D_3_ in fish has not been clarified. Both a non-photochemical pathway and a photochemical pathway for vitamin D_3_ synthesis in fish are doubted (Mattila et al., 1997). The latter due to limited UVB-light in their natural habitats combined with low amounts of 7-dehydrocholesterol in fish skin (Bills, 1927; Sugisaki et al., 1974; Takeuchi et al., 1991; Sunita Rao and Raghuramulu, 1996b; Rao and Raghuramulu, 1997). Evidence, on the other hand, exist that microalgae as the basis of the food chain is the origin of the high content of vitamin D_3_ in fish (Takeuchi et al., 1991; Sunita Rao and Raghuramulu, 1996a). However, data for vitamin D in algae are limited and not consistent (De Roeck-Holtzhauer et al., 1991; Takeuchi et al., 1991; Sunita Rao and Raghuramulu, 1996a; Brown et al., 1999). Takeuchi et al. (1991) found significant amounts of vitamin D_2_ (1.9–4.3 μg/100 g), vitamin D_3_ (5.0–15 μg/100 g) and their provitamins (260–1450 μg/100 g) in microalgae. Sunita Rao and Raghuramulu (1996a) also reported high concentrations of ergosterol (390 μg/100 g), 7-dehydrocholesterol (2400 μg/100 g), vitamin D_2_ (5.3 μg/100 g) and vitamin D_3_ (80 μg/100 g) in freshwater microalgae. The content of vitamin D_2_ and vitamin D_3_ in four Australian microalgae studied by Brown et al. (1999) were in all cases below the detection limit (35 μg/100 g) of the method used. De Roeck-Holtzhauer et al. (1991) studied vitamin D_2_ in several algae including the macroalgae *Sargassum multicum* and found very high amounts (90–3900 μg/100 g). No studies on vitamin D_3_ in macroalage have been performed. Both vitamin D_2_ and vitamin D_3_ are available for fish in their diet, but vitamin D_2_ is almost absent in fish (Lock et al., 2010). This suggests that the bioavailability of vitamin D_2_ is lower than for vitamin D_3_ (Andrews et al., 1980; Barnett et al., 1982; Takeuchi et al., 1991).

Microalgae usually live at the surface of the water and vitamin D is probably synthesized by sun exposure of provitamins D (Takeuchi et al., 1991). Takeuchi et al. (1991) observed that microalgae caught in August were higher in vitamin D than in October and December, which supports that vitamin D is synthesized from sun exposure of provitamin D. To synthesize vitamin D_3_ by UVB exposure, microalgae should be able to synthesize 7-dehydrocholesterol if using the same pathway as vertebrates. However, the sterols found in microalgae display a great diversity as may be expected from the large number of classes and species combined with a long evolutionary history (Volkman, 2003). Red algae (*Rhodophyta*) primarily contain cholesterol, although several species contain large amounts of desmosterol. Fucosterol is the dominant sterol of brown algae (*Phaeophyta*) (Patterson, 1971). Generalizations about the sterols in most other algae, e.g., diatoms (*Bacillariophyta*) and green algae (*Chlorophy*ta) cannot be made as they are much more varied (Patterson, 1971). The most common sterol in diatoms are 24-methylcholesta-5,24(28)-dien-3β-ol, but cholesterol and sitosterol are also common (Rampen et al., 2010). The green algae are very variable, they contain significantly amounts of 24-ethyl sterols (Volkman, 2003), but also cholesterol and ergosterol (Patterson, 1974). Microalgae are an extremely diverse group, as also seen from the large variability in the sterol content. It is, therefore, difficult to make any conclusions about algae's production of vitamin D_2_ and vitamin D_3_. Species differences and geographic differences may be expected.

## Analytical methods to study the vitamin D forms in plants

Research into vitamin D in plants is limited, presumably due to limitations in selectivity and sensitivity of the analytical methods available. Determination of vitamin D in food has always been a challenge due to low amounts of vitamin D combined with the existence of multiple vitamin D active compounds. Plants are a complex matrix, which makes the analysis of vitamin D even more challenging. Selective and sensitive methods are, therefore, a prerequisite. Each step in the analytical methods used in the research of vitamin D in plants will be discussed in the following chapter.

### Biological methods for vitamin D

The official method for vitamin D was for many years the line test using animals. Either a rat or a chicken was put on a vitamin D deficient diet until the animal developed rickets. Afterwards, they were fed plants or plant extracts and it was estimated to which extent the rickets were cured (Wallis, 1938, 1939; Moore et al., 1948; Thomas and Moore, 1951; Thomas, 1952; Keener, 1954; Henry et al., 1958; Wallis et al., 1958). This method is time-consuming as it takes 5 weeks, and precision and accuracy may be discussed. However, an advantage of this method may be that the amount of quantified vitamin D corresponds to the total vitamin D activity independent of the specific metabolites and their difference in activity. The interest in the 1,25(OH)_2_D metabolite initiated the use of more specific methods utilizing that a high strontium intake by chickens block the conversion of 25OHD to 1,25(OH)_2_D by suppressing 1α-hydroxylase activity (Wasserman, 1974; Weissenberg et al., 1989). This means that the inhibitory effect of strontium can be overcome by the administration of 1,25(OH)_2_D, but not by 25OHD and vitamin D. Studies of calcium absorption in nephrectomized rats that possess a suppressed 1α-hydroxylase activity (Walling and Kimberg, 1975) and assays with organ-culture systems such as cultured duodenum have also been used to study 1,25(OH)_2_D specifically in plants (Corradino and Wasserman, 1974). However, the biological activity measured in these methods could be due to other compounds that interfere with vitamin D metabolism, calcium absorption or to other compounds present, e.g., calcium and phosphorus that increase or inhibit the activity of vitamin D. More specific methods are, therefore, needed to study vitamin D and its metabolites in details.

### Chemical methods for vitamin D—sample preparation

Proper sample preparation is crucial for reliable analysis and should optimally release all vitamin D active compounds. Glycosylation and acetylation is general metabolic processes that occur in plants and vitamin D and related compounds are expected to be found as glycosides, esters and acetylated glycosides (Figure 3). Saponification followed by liquid-liquid extraction is typically used to liberate esters, where cold saponification is preferred over hot saponification due to reversible and temperature-dependent equilibration between vitamin D and pre-vitamin D (Buisman et al., 1968; Hanewald et al., 1968). However, saponification fails to hydrolyze the bond between vitamin D and the carbohydrate moiety in the glycosides. Both direct and indirect analysis (with or without hydrolysis) can be used for glycosides (Van Hoed et al., 2008). Direct analysis is fast, as a sample preparation step is omitted, but complicated as the needed conjugated standards is non-available. For indirect analyses, acid hydrolysis has been used to release glycosidic forms (Toivo et al., 2001; Liu et al., 2007; Nyström et al., 2007). Acid hydrolysis is typical performed under relatively harsh conditions, e.g., 60 min at 80°C with 6 M ethanolic hydrochloric acid solution (Kamal-Eldin et al., 1998; Toivo et al., 2001; Nyström et al., 2007). This is not optimal due to risk of isomerization of certain sterols (Kamal-Eldin et al., 1998) including 5,7-dienes as 7-dehydrocholesterol (Dolle et al., 1988) as well as vitamin D_3_ (Jin et al., 2004). An alternative to acid hydrolysis is the gentler enzymatic hydrolysis. Kesselmeier et al. (1985) used β-glucosidase in the hydrolysis of steryl glycosides in oat leaves and seeds, but other researchers have not been successful in repeating these results (Moreau and Hicks, 2004; Nyström et al., 2008). A hypothesis is that the observation by Kesselmeier et al. (1985) may be due to impurities of minor enzymes rather than the actual β-glucosidase, whereas similar secondary activities not are present in modern highly purified enzyme preparations (Moreau and Hicks, 2004; Nyström et al., 2008).

The extraction of liberated vitamin D compounds from the non-saponifiable matter is usually performed by liquid/liquid extraction using non-polar organic solvents (CEN, 2008). Further clean-up of the extracts is usually needed to remove interfering compounds and to avoid contamination of the analytical column by other co-extracted substances, e.g. chlorophyll and other lipophilic pigments (Jäpelt et al., 2011b). Combinations of column chromatography or/and preparative HPLC have been used for purification of plant extracts before vitamin D analysis (Rambeck et al., 1979; Esparza et al., 1982; Morris and Levack, 1982; Prema and Raghuramulu, 1994; Curino et al., 1998, 2001; Skliar et al., 2000). However, fractionation by column chromatography is time-consuming and not suitable for routine analysis and has recently been replaced by solid phase extraction (SPE) (Jäpelt et al., 2011a,b).

If total vitamin D activity is required, the sum of vitamin D and any other metabolites that may have vitamin D activity must be quantified. The hydroxylated metabolites have higher polarity than vitamin D, but despite the difference in polarity are vitamin D and 25OHD extracted in the same run (Mattila et al., 1995a; Jakobsen et al., 2004). Only few studies have included quantification of 1,25(OH)_2_D in food (Kunz et al., 1984; Takeuchi et al., 1988; Montgomery et al., 2000). These studies omitted the saponification step, which seems to question whether conjugated forms of 1,25(OH)_2_D will be quantified. Our recent study, included saponification in the analysis of 1,25(OH)_2_D in plant material, but poor extraction efficiency from the non-saponifiable matter was observed, which increased the detection limit (Jäpelt et al., 2012). Therefore, optimization of the extraction procedure is needed.

### Quantification of vitamin D forms

An internal standard is essential for quantification of vitamin D due to reversible isomerization with the corresponding previtamin D (Schlatmann et al., 1964). An internal standard is also needed to eliminate analytical errors due to losses of vitamin D during sample preparation and to compensate for signal variation if using mass spectrometry (MS) detection (Dimartino, 2007). Vitamin D_2_ and vitamin D_3_ are chemically very similar and vitamin D_2_ has been used as internal standard when determining vitamin D_3_ and *vice versa*. However, this is not the best approach when vitamin D_2_ and vitamin D_3_ occur simultaneously as could be the case in plants (Horst et al., 1984). For quantification by MS isotopic labeled compounds are ideal internal standards, because of the complete resemblance with the analyte.

### Separation and detection principles for vitamin D and its sterol precursors

#### Gas chromatography flame ionization detection (GC-FID) and gas chromatography mass spectrometry (GC-MS)

Sterols act as precursors of vitamin D so sterol analysis is essential to investigate the biosynthesis of vitamin D in plants. Sterols are typically measured by gas chromatography (GC) as trimethylsilyl (TMS) ether derivates (Piironen et al., 2000), which are detected either by flame ionization detection (FID) (Phillips et al., 2005; Brufau et al., 2006; Liu et al., 2007) or MS (Toivo et al., 2001; Nyström et al., 2007). GC was also the first chromatography principle used to replace the biological assay for analysis of vitamin D (Bell and Christie, 1973), but while GC is a good separation method for sterols it is not the best choice for vitamin D. Vitamin D undergoes thermal cyclization in a GC split/splitless injector (>125°C) resulting in formation of the corresponding pyro and isopyro compounds with a concomitant decrease in sensitivity (Yeung and Vouros, 1995). However, early studies did use GC for identification of vitamin D_3_ in plants (Rambeck et al., 1979; Suardi et al., 1994).

#### High performance liquid chromatography with UV detection (HPLC-UV)

HPLC with UV detection (265 nm) is used in official methods for vitamin D in food (Staffas and Nyman, 2003; CEN, 2008) and has also been used in recent studies on vitamin D in plants (Prema and Raghuramulu, 1994, 1996; Aburjai et al., 1996, 1997, 1998; Curino et al., 1998, 2001). Nevertheless, these methods are laborious as high degree of purification of the extracts is needed. Analysis of vitamin D in complex matrices such as plants is especially challenging due to co-eluting interferences.

GC is generally considered superior to HPLC for sterol analysis (Lagarda et al., 2006), but introduction of columns with particle sizes of 1–2 μm improve resolution of co-eluting sterols and may bring HPLC ahead of GC (Lu et al., 2007). Furthermore, HPLC have compared to GC the advantage of analysis without derivatization and gentler conditions suitable for thermally unstable sterols. Even though HPLC may be combined with UV for detection of sterols (Careri et al., 2001; Sanchez-Machado et al., 2004), this is not the most sensitive method, as sterols adsorb UV between 200 and 210 nm where most organic solvents have low transparency.

#### Liquid chromatography mass spectrometry (MS, LC-MS, LC-MS/MS)

Detection of vitamin D by MS detection is challenging due to low ionization efficiency. The most used ionization source for LC-MS is ESI, which works best when the analyte already is in its ionic form in solution (Cech and Enke, 2001). The ionization efficiency of vitamin D and its sterol precursors are as a result low in most ESI methods (Dimartino, 2007). APCI is a much more efficient ionization technique for neutral and apolar substances such as vitamin D and has been used several times for vitamin D analysis (Dimartino, 2007; Byrdwell, 2009; Jäpelt et al., 2011b). Atmospheric pressure photoionization (APPI) is another ionization method suitable for lipophilic compounds, which also has been used for detection of vitamin D (Soldin et al., 2009). MS has been used for identification of vitamin D_3_ in plants in several studies, but in most cases not coupled to liquid chromatography (LC) (Aburjai et al., 1996; Curino et al., 1998; Skliar et al., 2000). We recently used liquid chromatography tandem mass spectrometry (LC-MS/MS) for selective detection of vitamin D in various plant matrices (Jäpelt et al., 2011a,b). LC-MS/MS improves both selectivity and sensitivity compared to LC-MS in particular by using selected reaction monitoring (SRM). In SRM both a precursor and a product ion is selected, which reduce background noise resulting in a good signal to noise ratio. SRM increases selectivity, but more than one transition is needed for reliable confirmation, which preferable is combined with other evidence such as relative intensities of product ions in the mass spectra, accurate mass, retention time and peak shape to positively identify the compound as vitamin D (Jäpelt et al., 2011b). LC-MS and LC-MS/MS have also been used several times for analysis of sterols in plant matrices (Mezine et al., 2003; Rozenberg et al., 2003; Ruibal-Mendieta et al., 2004; Cañabate-Díaz et al., 2007; Lu et al., 2007; Jäpelt et al., 2011b). To study vitamin D and its sterol precursors in plants LC-MS/MS is the method of choice. However, a significant challenge is that the content of various sterols span several orders of magnitude. The major sterols such as sitosterol and campesterol is between 10 and 200 μg/g fresh weight, whereas minor sterols and vitamin D_3_ are present at less than 0.1 μg/g fresh weight (Jäpelt et al., 2011b; Schrick et al., 2011). This requires a huge dynamic range of the analytical method or fractionation of the extracts.

#### Nuclear magnetic resonance (NMR)

Nuclear magnetic resonance (NMR) is a powerful tool for structure elucidation and identification and offer valuable information in addition to UV and MS detection. NMR can discriminate between compounds that only differ in terms of local chemical environment, e.g., compounds with same mass, but different locations of functional groups. However, in general NMR analyses require extensive purified samples, and possess low sensitivity (Eisenreich and Bacher, 2007). Nevertheless, ^1^H NMR has been used for identification of vitamin D_3_ in plants, but extraction of as much as 2 kg fresh plant leaves was required (Aburjai et al., 1998).

### Analytical methods for quantification of hydroxylated metabolites of vitamin D

Analysis of the hydroxylated metabolites of vitamin D represents a challenge because they exist in even lower concentrations than vitamin D (Aburjai et al., 1996, 1998; Prema and Raghuramulu, 1996). They have been detected in plants using both protein-binding assays (Skliar et al., 2000; Curino et al., 2001) and chemical methods such as HPLC with UV detection (Aburjai et al., 1998, 1996; Prema and Raghuramulu, 1994, 1996) and MS detection (Jäpelt et al., 2012). Protein-binding assays, including RIA (radioimmunoassay) and RRA (radioreceptor binding assay), are widely used in clinical laboratories for analysis of 25OHD and 1,25(OH)_2_D in serum due to the simplicity (Hollis and Horst, 2007). RIAs are commercially available and have been used for extracts and cell cultures of *S. glaucophyllum* and *C. diurnum* (Weissenberg et al., 1988; Gil et al., 2007). RRA has been applied for identification of 1,25(OH)_2_D_3_ in *S. glaucophyllum* (Curino et al., 2001) and *Nicotiana glauca* Graham (Skliar et al., 2000). However, the lipophilic nature of vitamin D makes it difficult to analyze in any protein-binding assay due to solubility problems (Hollis and Horst, 2007). Matrix effects are also common due to interfering compounds found in the assay tube but not in the standard that compete with binding to the protein. The most common chemical detection principle used for the detection of the hydroxylated metabolites in plants has been HPLC-UV, but this is not totally specific. Specific quantification of vitamin D metabolites can on the other hand be obtained by using MS methods. However, direct LC-MS/MS analysis of especially 1,25(OH)_2_D is challenging because of poor ionization efficiency, low concentration and an extensive product ion spectra by most soft ionization techniques (Aronov et al., 2008). Attempts to increase ionization efficiency have been reported several times mostly for serum samples, these include adduct formation (Kissmeyer and Sonne, 2001; Casetta et al., 2010), derivatization with Cookson-type reagents (Higashi and Shimada, 2004; Gao et al., 2005; Kamao et al., 2007; Aronov et al., 2008; Higashi et al., 2011) and microflow LC-MS together with derivatization (Duan et al., 2010). Microflow LC improve sensitivity 15-fold compared to normal LC, but has a small loading capacity that counteracts the sensitivity gain, especially when analyzing complex matrices (Duan et al., 2010). The advantage of using microflow LC may, therefore, be limited for analysis of plant extracts. Recently, LC-ESI-MS/MS in combination with Diels-Alder derivatization was used to study 25OHD_3_ and 1,25(OH)_2_D_3_ in the leaves of *S. glaucophyllum, S. lycopersicum* and *C. annuum* (Jäpelt et al., 2012).

## Concluding remarks

Vitamin D deficiency is a problem in populations with limited sun exposure where a dietary intake of vitamin D becomes essential. However, dietary recommendations for vitamin D are difficult to meet because few food items naturally contain vitamin D and it would, therefore, be valuable to increase the food sources of vitamin D in the human diet or to optimize the content by bio-fortification. Traditionally, only animal products have been considered a source of vitamin D_3_, but today we know that vitamin D_3_ and its metabolites are formed in certain plants. Accordingly, fruits and vegetables have the potential to serve as a source of vitamin D. Especially, the *Solanaceae* family contains high amounts of vitamin D_3_, which is of special interest considering the importance of this family in human nutrition. The *Solanaceae* family includes important vegetables such as potato, tomato and pepper all of which have been found to contain vitamin D_3_. Our current knowledge is limited to the content in leaves, but future investigation will elucidate if also the edible portions contain vitamin D_3_. It would be valuable to screen a variety of crops and vegetables for vitamin D, but to carry out a larger screening development of less time-consuming and preferably more sensitive analytical methods are needed. A further challenge is to improve methods to study and quantify vitamin D conjugates in details.

Planktonic microalgae, inhabiting the sea, are another large group of photosynthetic organisms that contain vitamin D. Microalgae are, as part of the aquatic food chain, identified as a source of vitamin D for fish. Currently, the world's wild fish stocks are being overexploited and there has been a growth in the aqua-culture industry. The current trend is to replace fish meals or fish oil partly by vegetable feed substitutes when feeding cultured fish will reduce the content of vitamin D compared to wild fish (Bell and Waagbø, 2008). Microalgae with a high natural amount of vitamin D may be used as a natural vegetable form for the bio-fortification of aqua-cultured fish.

Basic knowledge about the biosynthesis of vitamin D_3_ in photosynthetic organisms is still lacking and any increase in our knowledge will help us to manipulate the content to produce plants with a higher natural amount of vitamin D_3_. Vitamin D_3_ is only synthesized in minute amounts, which makes it challenging to study the pathways and enzymes involved. However, it also means that even small changes in vitamin D_3_ can have a significant impact on human health. Biosynthesis of 24-desmethylsterols in plants is complex and poorly understood and makes the final goal to produce plants with a higher natural amount of vitamin D_3_ a great challenge. Currently, the key biosynthetic steps and the enzymes involved are unknown. These need to be identified before we even can begin to modify the content of vitamin D_3_ in plants. In the present review, a hypothetical biosynthetic pathway for 7-dehydrocholesterol and cholesterol is presented. The steps catalyzed by SMT1 and DWARF5 seem to be promising targets to manipulate the level of 7-dehydrocholesterol in plants. A block in SMT1 will force the biosynthetic pathway in the direction of 7-dehydrocholesterol and cholesterol. Further increase in 7-dehydrocholesterol can probably be achieved by a block in Δ^5, 7^-sterol-Δ^7^-reductase (DWARF5). However, any increase in provitamin D_3_ should be viewed in the context of the overall changes in the metabolic profile and a significant challenge will be selective to accumulate vitamin D_3_ in edible organs such as fruits, without affecting plant growth and the development of the plant and consequently yields. An important thing to consider before putting a lot of energy into producing plants with a high amount of vitamin D is the bioavailability, as low bioavailability of vitamin D from plants may diminish the potential of plants as a new vitamin D source.

### Conflict of interest statement

The authors declare that the research was conducted in the absence of any commercial or financial relationships that could be construed as a potential conflict of interest.
